# Prevalence of depressive symptoms among non insulin treated Greek type 2 diabetic subjects

**DOI:** 10.1186/1756-0500-1-101

**Published:** 2008-10-28

**Authors:** Alexios Sotiropoulos, Athanasia Papazafiropoulou, Ourania Apostolou, Anthi Kokolaki, Aristofanis Gikas, Stavros Pappas

**Affiliations:** 1 3rd Department of Internal Medicine and Center of Diabetes, General Hospital of Nikaia "Saint Panteleimon" – Piraeus, Greece; 2 Department of General Practice, Health Centre of Kalivia, Kalivia-Lagonisi, Athens, Greece

## Abstract

**Background:**

Depression is common among diabetic subjects. We conducted the present study to estimate the prevalence of depression in subjects with type 2 diabetes (T2D) in Greece.

**Methods:**

The study sample consisted of 320 T2D subjects without overt macrovascular disease attending the diabetes outpatient clinic of our hospital, from June 2007 to December 2007. Depressive symptoms were measured using the 21-item Beck Depression Inventory, modified for use in diabetic subjects.

**Results:**

Of the study subjects 107 (33.4%) reported elevated depressive symptoms. More women than men with diabetes reported symptoms of depression (48.4% vs. 12.7%, *P *< 0.001). In the female study group, depressive symptoms were correlated with HbA_1c _(*P *= 0.04), and duration of diabetes (*P *= 0.004). In the male study group, univariate linear regression analysis showed no significant relationships between depressive symptoms and the testing variables.

**Conclusion:**

The prevalence of depression in Greek T2D subjects is high. Diabetic female subjects showed increased levels of depressive symptoms compared with male subjects. Independent risk factors of depressive symptoms in diabetic female subjects were diabetes duration and glycemic control.

## Background

Depression is common among people with diabetes [1], especially among those with diabetic complications [2]. Depression has been associated with poor adherence to medication [3], poor glycemic control [4], as well as with the development of diabetic complications [5] and increased mortality [6]. Several studies focusing on the prevalence of depression in people with diabetes have been done, showing different depression rates [7,8].

The available data regarding the prevalence of depression among subjects with type 2 diabetes (T2D) in Greece are limited. We, therefore, conducted the present study to evaluate the prevalence of depressive symptoms as well as gender differences in subjects with T2D. In addition, the relationship between depressive symptoms and glycemic control was examined.

## Methods

### Population

The study sample consisted of 320 subjects (age 35–70 years) with T2D attending the diabetes outpatient clinic of our hospital, from June 2007 to December 2007. T2D was diagnosed according to the American Diabetes Association criteria [9]. Subjects with clinically apparent macrovascular disease (coronary, peripheral and carotid artery disease or stroke) and on insulin treatment were excluded since it has been shown that they have high prevalence of depressive symptoms [4,5]. The study protocol was approved by the Scientific and Ethical Committee of the General Hospital of Nikaia.

A detailed medical history was obtained in all study subjects and a full clinical examination was performed. Glycemic control was assessed by measuring of HbA_1c _(non-diabetic reference range of 4.1–6.0%), which is reported to be a reliable indicator of blood glucose level for the last three months prior to testing [10]. Body weight with subjects in light clothing without shoes and height was measured and body mass index (BMI) was calculated.

### Assessment of depressive symptoms

Depressive symptoms were measured using the 21-item Beck Depression Inventory (BDI), modified for use in diabetic subjects, as described in detail elsewhere [11-13]. Each of the 21 items on the BDI measures the presence and severity of a symptom of depression by requiring a self-rating from 0 to 3. A score is determined by summing the ratings for the individual items [11-13]. The standard cut-offs are as follows: 0–9 indicates that a person is not depressed, 10–18 indicates mild-moderate depression, 19–29 indicates moderate-severe depression and 30–63 indicates severe depression [11-13]. According to the above scales, we considered as a cutt-off point of elevated depressive symptoms the total score ≥ 19.

The BDI is designed to examine both somatic and cognitive aspects of depression [11-13]. The BDI has been used, apart from the assessment of the severity of known depression, for screening purposes. Studies have showed that BDI is an effective screening test for depression in diabetic subjects [12,13].

### Statistical Analyses

The following tests were used for the statistical analysis of the data: the student's *t *test was used to assess differences in continuous variables, while the χ^2 ^test was used for categorical data. Linear regression analysis was used to assess the relationship between depressive symptoms and the parameters of metabolic control. P-values < 0.05 were considered statistically significant.

## Results

**Figure 1:**
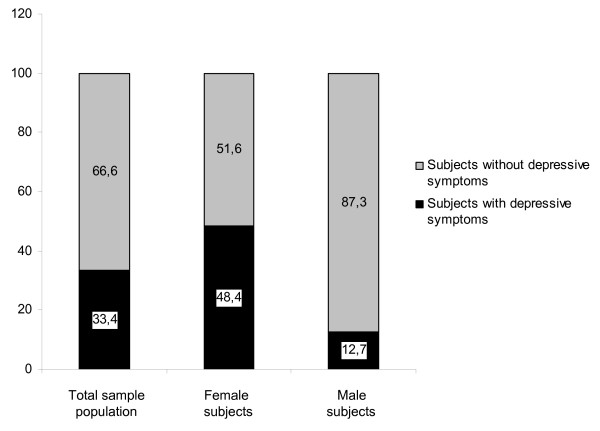
The depression prevalence in the total sample population and according to gender (Data are shown as %).

The demographic characteristics of the study population are shown in Table 1. Of the study subjects 107 (33.4%) reported elevated depressive symptoms. Female diabetic subjects showed more often depressive symptoms than male diabetic subjects (48.4% vs. 12.7%, *P *< 0.001) (Figure 1).

**Table 1:** Demographic and clinical characteristics of the study patients.

	Males	Females	P-value
n (%)	134 (41.9)	186 (58.1)	-
Age (years)	62.8 ± 6.8	63.6 ± 7.6	NS
BMI (kg/m^2^)	28.9 ± 4.9	31.2 ± 5.4	< 0.001
Duration of diabetes (years)	10.5 ± 6.1	12.3 ± 7.2	0.03
HbA_1c _(%)	7.2 ± 1.3	7.7 ± 1.7	0.001
Retinopathy (yes) n (%)	14 (10.4)	23 (12.4)	NS
Neuropathy (yes) n (%)	13 (9.7)	19 (10.2)	NS
Nephropathy (yes) n (%)	2 (1.5)	4 (2.2)	NS
Depression (yes) n (%)	17 (12.7)	90 (48.4)	< 0.001

Data are shown as mean ± SD or as n (%).

In the female study group, univariate linear regression analysis showed significant relationships between depressive symptoms and HbA_1c _(*beta *= 0.11, *P *= 0.04), and duration of diabetes (*beta *= 0.16, *P *= 0.004). No significant associations were found between depressive symptoms and age, BMI, as well as microvascular complications.

In the male study group, univariate linear regression analysis showed no significant relationships between depressive symptoms and the testing variables.

## Discussion

The present study showed that the prevalence of depression in a sample of a Greek T2D people was high. The data regarding the prevalence of depression in T2D subjects in our country are limited. A previous study reported the frequency of depressive symptoms only in type 1 diabetic patients [14] and another study in elderly subjects with diabetes in primary health care [15]. A recent study in a small sample of elderly diabetic subjects showed no relationship between diabetes and depression [16].

There is evidence that diabetic people are twice as likely to have depression compared with nondiabetic persons [1,17]. Approximately 30% of diabetic subjects have clinically relevant depression [1,17]. However, there are studies showing conflicting results. A study in newly identified T2D subjects reported no association between diabetes and depression [18]. Other studies showed that the risk of depression in diabetes increases only when comorbid diseases was present [19,20]. In the present study T2D subjects with macrovascular complications were excluded and therefore the observed prevalence of depression among T2D subjects is independent of the presence of comorbid diseases.

The prevalence of depression was significantly higher in females with diabetes compared with males, confirming previous results [21-24]. This pattern is similar to reporting rates in the general population as well as other medical conditions [25-27]. It is known that major depression occurs twice as frequently in women than in men [28] and seems to be influenced by oestrogen levels [29]. A recent study suggested that depressive symptoms are associated with a modest increase in the risk of T2D in female subjects [30].

In the female study group depressive symptoms were associated with poor glycemic control and duration of diabetes, confirming previous reports [21-23]. A meta-analysis of 28 studies concluded that depression was associated with hyperglycemia in patients with T2D [31]. On the contrary, a study showed an association between depression and HbA_1c _levels only in type 1, but not in T2D patients [24]. Another studies showed that depression does not influence metabolic control in diabetic people [32] and that depression appears to be stronger in men than in women [33].

In this study, we did not find any relationship between depression and BMI, in contrast to two studies showing higher BMI in depressed than non depressive diabetic subjects [1,34]. In addition, no relationship was found between depressive symptoms and microvascular complications, although previous studies have reported the opposite [35,36].

Our study has some limitations. Data were collected from an outpatient diabetic clinic. A possible selection bias could not be excluded since the more concerned diabetic patients might seek a specialized diabetes care. Our assessment of depression was based on self-report of symptoms using a validated instrument, not on the more accurate clinical diagnostic interview. Furthermore, data on demographic characteristics of the study subjects were not available. Studies have showed that depressive symptoms are higher in diabetic people with lower income as well lower education level [37]. Finally, diabetic subjects on insulin therapy and with macrovascular disease were excluded and therefore our results can not be extrapolated to the diabetic population.

In conclusion, the present study showed that the prevalence of depression in Greek T2D subjects without overt macrovascular complications is comparable to findings from the recent literature. Diabetic female subjects showed increased levels of depressive symptoms compared with male subjects. Independent risk factors of depressive symptoms were diabetes duration and glycemic control.

## Competing interests

The authors declare that they have no competing interests.

## Authors' contributions

All authors participated in the collection, analysis, interpretation of data and writing of the paper.
